# Resident Support for the Federally Mandated Smoke-Free Rule in Public Housing: 2018–2022

**DOI:** 10.3390/ijerph21010102

**Published:** 2024-01-17

**Authors:** Craig T. Dearfield, Margaret Ulfers, Kimberly Horn, Debra H. Bernat

**Affiliations:** 1 Department of Epidemiology, The Milken Institute School of Public Health, The George Washington University, Washington, DC 20052, USA; mulfers@gwu.edu (M.U.); dbernat@gwu.edu (D.H.B.); 2 Department of Population Health Sciences, Virginia Tech-Carilion Fralin Biomedical Research Institute, Roanoke, VA 24016, USA; kahorn1@vt.edu

**Keywords:** tobacco, public policy, policy surveillance, housing, evaluation

## Abstract

This study examines support for the Department of Housing and Urban Development’s (HUD) mandatory smoke-free rule up to four years post-rule among smokers and non-smokers. A repeated cross-sectional design was used where District of Columbia public housing residents aged 18+ (*n* = 529) completed surveys during three time points: July 2018 (pre-rule), November 2018–March 2020 (post-rule), and September 2020–December 2022 (post-rule + COVID-19). Full support for the rule was indicated by agreeing that smoking should not be allowed in all indoor locations and within 25 feet of buildings. Descriptive statistics showed significant differences in support across time for smokers (5.3%, 30.7%, and 22.5%, respectively) and similar support across time for nonsmokers (48.2%, 52.2%, and 40.0%, respectively). In unstratified regression analysis, pre-rule support was lower than when the rule was in effect (aOR = 0.47, 95% CI = 0.25, 0.90), and tobacco users were less likely to support the rule (aOR = 0.34, 95% CI = 0.23, 0.50). Stratified logistic regression results showed that pre-rule support was lower among smokers compared to post-rule support (aOR = 0.14, 95% CI = 0.03, 0.59); support among nonsmokers did not vary by time. Findings overall indicate low support for the smoke-free rule up to 4 years post-implementation. Engaging residents with the rule and promoting health and well-being may further enhance policy effectiveness and acceptance.

## 1. Introduction

The Department of Housing and Urban Development (HUD) implemented a mandatory smoke-free rule for all public housing agencies (PHAs) in the US beginning in July 2018 [1]. Current evidence suggests that legislative smoking bans improve population health by reducing the prevalence of smoking-related illnesses and reducing secondhand smoke exposures [2]. The HUD smoke-free rule prohibits the use of lit tobacco products in all indoor areas, including individual units, and outdoors within 25 feet of Housing Authority buildings [1].

Many evaluations of resident support for a smoke-free housing rule were conducted based on voluntary rules, either restricting smoking in the entire building or individuals adopting smoking restrictions for their homes [3]. Ahead of the HUD mandatory smoke-free rule, a review assessing support for smoke-free policies among residents of any type of multiunit housing (including public housing and subsidized housing residents) found approximately 80% of nonsmokers supported the policies and about 20% of smokers supported them across several settings [3]. The review concludes that the concerns and support of residents should be an important part of implementing these rules in order to help those communities successfully see the benefits of the rules. These findings are similar to other prior research that consistently shows non-smokers support smoke-free housing rules at higher rates than smokers, often at double the level [4,5,6,7,8,9,10,11,12].

One study of the mandatory HUD rule to date provided quantitative findings that were consistent with these pre-rule estimates. This study examined resident perceptions of the rule in the four months prior to rule implementation and 12 months after the rule went into effect and found approximately 95% of New York City Housing Authority (NYCHA) non-smoking residents supported the rule at both pre- and post-test times [13].

This study addresses the dearth of studies on support for the mandatory HUD smoke-free rule. Other studies to date examining the support among public housing residents primarily address voluntary rules in specific housing authorities or buildings [3,8,12], hypothetical smoke-free rules [9,10,11], or were conducted in the first year following implementation in July 2018 [13]. Longer-term follow-up will allow for the assessment of trends in support over time, which is especially important as previous results have shown support for smoke-free rules generally increases over time [7]. Additionally, quantitative examinations of support among public housing residents following the mandatory HUD rule are lacking, with most studies consisting of qualitative results [13,14,15]. While this presents a detailed picture of residents’ perceptions of the rule, it does not directly compare to prior quantitative evaluations of support among residents of public housing, residents of subsidized housing, and other residents in multiunit buildings. Studies of support for smoke-free rules also disproportionately examine the feelings of non-smoking residents [3]. The purpose of this paper is to examine support for the HUD federally mandated smoke-free rule before and after implementation by smoking status.

## 2. Methods

### 2.1. Procedures

Residents aged 18 and older from 15 District of Columbia Housing Authority (DCHA) properties were eligible to participate. Of the fifteen properties, seven served senior or disabled residents, and eight served families and residents of all types. Participants were recruited through resident meetings, fliers, and word-of-mouth. Data were collected at three time points (T) between July 2018 and December 2022. Data were collected in July 2018 (T1) to assess support before the rule, November 2018 to March 2020 (T2) to assess support post-rule, and September 2020 to December 2022 (T3) to assess longer post-rule support, which occurred during the COVID-19 pandemic. Residents could participate in multiple waves.

The study design was a repeated cross-sectional design. Of the 662 participants in the total study, 133 residents participated more than once. The analysis was limited to the 529 residents who answered the survey once during the study and who had complete data for rule support and all predictor variables.

Data were collected in-person on DCHA property using computer-assisted self-interviewing software from a convenience sample of residents (T1 and T2) and via a telephone survey of residents who agreed to participate in later data collection activities or new residents referred to the study by DCHA personnel and other residents (T3). The George Washington University Institutional Review Board approved all study procedures (#180523). Residents provided signed written consent to participate in the study during in-person events. The consent documents were distributed, and the research staff read the documents aloud to residents. Residents provided verbal consent to participate in the study during telephone interviews. Study staff read the consent documents to residents who participated in telephone data collection. During all data collection activities, residents provided written or verbal consent to participate in follow-up data collection.

### 2.2. Measures

Resident Characteristics: All data were self-reported. Participants indicated their sex (male/female) and age (in years). Participants’ ages were recoded into a dichotomous variable to indicate if they were aged 18 to 64 or 65 and older. For race data, participants were asked to check all options that apply to themselves among American Indian or Alaska Native, Asian, Black or African American, Native Hawaiian or Other Pacific Islander, White, and Other.

Residents reported the highest grade/level of schooling completed. This information was recoded into a dichotomous variable indicating high school completion, more education, or less than completion of high school, and a General Equivalency Diploma (GED). Residents with a GED were categorized with those who had not completed high school because prior studies have shown those with a GED are more like those who do not have a high school diploma regarding health status [16].

Tobacco Use: Residents reported whether they had used the following tobacco products in the past 30 days (yes/no): cigarettes, cigars, little cigars and cigarillos, smokeless tobacco, hookah, and e-cigarettes. Tobacco use was recoded into a dichotomous variable indicating whether the resident used a tobacco product affected by the rule (cigarettes, cigars, little cigars and cigarillos, and hookah). No resident used only e-cigarettes in the prior month at the time of their survey, and two residents indicated using only smokeless tobacco in the prior month. These two residents were treated as non-users of tobacco products affected by the rule.

Support for the Smoke-free Rule: Participants indicated whether lit tobacco products should be able to be used in five locations prohibited by the rule: individual units, balconies, common areas, administrative offices, and within 25 feet of housing property. Full support (yes/no) for the smoke-free rule was indicated by agreeing that smoking should not be allowed in all five locations.

### 2.3. Data Analytic Strategy

Descriptive statistics were calculated for all study variables, and chi-square analyses were conducted to assess differences in resident characteristics across time points. Over 85% of residents identified as non-Hispanic Black/African American, so race/ethnicity variables were omitted from all analyses due to low variability. Logistic regression analyses were used to compare rule support at three time points, controlling for sex, age, and education. All analyses were performed in SAS version 9.4.

## 3. Results

### 3.1. Demographics

Table 1 shows the characteristics of the sample by time. Most residents provided data during the intervention period (69.8%, *n* = 369), with 12.3% (*n* = 65) providing data during the pre-rule period and 18.0% (*n* = 95) providing data during COVID-19. Most residents were aged 18–64 (66.5%, *n* = 352) and female (61.6%, *n* = 326). Slightly over one-half (56.5%, *n* = 299) of the residents had a high school diploma or more schooling. Slightly over one-third of all residents across time points reported that smoking should not be allowed in any of the areas banned by the federally mandated rule (37.4%, *n* = 198). About one-half of the residents were tobacco non-users (49.5%, *n* = 262). Results show significant differences in resident characteristics, indicating they must be included as covariates in regression models.

Table 1 also shows chi-squared test results. The highest proportion of residents aged 18–64 provided data during the pre-rule period (83.1%, *n* = 54). Additionally, the proportion of females was higher during pre-rule and COVID-19 (76.9%, *n* = 50; 72.6%, *n* = 69), and the proportion of residents with a high school diploma or more education was highest during COVID-19 (70.5%, *n* = 67). Support for the rule in chi-square tests was highest during implementation (41.2%, *n* = 152). There were no differences in the proportion of smokers by time point. Chi-squared test results indicate support among smokers significantly changed over time, from 5.3% (*n* = 2/38) during the pre-rule period to 30.7% (*n* = 58/189) during implementation and 22.5% (*n* = 9/40) during COVID-19. Support among non-smokers did not show a significant difference across time points in this test.

### 3.2. Regression Results

Table 2 shows the results of the regression models. In the unstratified model, support before the rule went into effect was significantly lower than when the rule was in effect (adjusted odds ratio [aOR] = 0.47, 95% confidence interval [CI] = 0.25, 0.90). Support from residents during COVID-19 was also lower than support during the implementation period, but not significantly lower. Tobacco users were significantly less likely to report support for the smoke-free rule than non-tobacco users (aOR = 0.34, 95% CI = 0.23, 0.50). Other demographic and resident characteristics of sex, age, and education were non-significant predictors in the unstratified model.

Additional models were conducted stratifying by tobacco use status. For the stratified models, none of the variables in the model for non-smokers significantly predicted support for the rule. The model for smokers showed a similar trend over the three data collection time points as the unstratified model. Support was significantly higher as the rule was implemented (aOR = 0.14, 95% CI = 0.03, 0.59), and lower during COVID-19, but not significantly lower. Other demographic and resident characteristics, such as sex, age, and education, were non-significant predictors in both stratified models.

## 4. Discussion

The current study provides longer-term follow-up results on the perceptions among public housing residents of the HUD smoke-free rule. Findings overall indicate low support for the smoke-free rule up to 4 years post-implementation. Total support varied between 23% and 41%, smoker support varied between 5% and 30%, and non-smoker support varied between 40% and 52%. These results represent lower values compared to evaluations of the HUD rule [13] and of other voluntary smoke-free housing rules [3,4,5,6,7,8,9,10,11,12]. In these studies, support for smoke-free housing rules among non-smokers is often as high as 95% [13], with many being over 60% [3]. Support among smokers is still commonly above 40% [3].

Additionally, findings indicate support for the smoke-free rule is associated with time since implementation and smoking status. Results showing smokers are less likely to support the rule are consistent with previous findings on this topic [3,4,5,6,7,8,9,10,11,12]. Descriptive and regression analyses additionally show support increases following implementation of the rule among smokers, which have not been identified in previous studies.

The pre-rule sample had the highest proportion of residents aged 18–64, and the proportion of females and individuals with a high school diploma or more education was highest during COVID-19, which could have implications for study results. Prior evidence also indicates support for smoke-free housing rules is associated with demographic characteristics. Specifically, support has been shown to be higher among those with higher education and who identify as females [11,17], as well as among those who identify as racial and ethnic minorities [17]. However, current analyses included these factors in adjusted regression models to minimize the impact of variations across data collection time periods, where they were non-significant predictors of rule support, so this imbalance was unlikely to change the results.

These findings are not consistent with prior studies that show support for smoke-free rules generally increases over time [7], potentially due to COVID-19-related restrictions. As reported in the literature, support for smoke-free rules typically increases with time, particularly within a year of implementing a smoke-free policy [7,18,19]. Other studies examining perceptions of the HUD smoke-free rule have shown that support is related to implementation processes, as support increases following the rule going into effect [13]. One potential pathway for this relationship is that residents began to see the positive influence of the rule in their community, and smokers adopted new habits to comply with the rules following implementation. Other long-term evaluations of the rule show decreased levels of secondhand smoke attributable to the rule [20], whereas shorter-term evaluations showed no impact on secondhand smoke [21,22,23]. But these perceptions were disrupted by COVID-19 when people were in their homes more, and smokers potentially lost the habits with which they complied with the rule. Additionally, informal compliance and enforcement processes where DCHA administrators walk the properties and talk with residents about smoking rules were stopped due to distancing restrictions, and these implementation procedures have been shown to be related to support for rule enforcement [13,14,15].

Additionally, one of the strengths of this study is that the analyses include a high proportion of tobacco users (over 50%), which has been identified as a limitation of some studies examining rule support [3]. Descriptive statistics indicate support among smokers in this sample is 20% lower among non-smokers, which is consistent with previous findings [3,8,9,10,11]. Smoking status also has an impact on the level of support over time. Stratified regression models show that smokers were significantly more likely to support the rule after it was implemented for a time, but their support fell during COVID-19 restrictions. Non-smokers have non-significant differences in support across the time points in this analysis, suggesting their support may be consistent over time.

Another strength is asking about support in multiple locations. This method has been used in other studies examining support across personal living spaces, common indoor areas, and outdoor areas [11,24], and represents an improvement from studies asking one question about personal living spaces [9,11]. This strength is based on asking residents about areas where smoking is specifically prohibited under the HUD rule.

The study’s findings underscore the importance of continuous education and reinforcement regarding smoke-free policies in public housing. There is a clear need for tailored interventions, especially given the varying support based on smoking status. The decline in support during the COVID-19 pandemic suggests that policies should be adaptable to unforeseen and anomalous circumstances and should employ diverse enforcement strategies. Engaging residents in decision-making and promoting holistic health can further enhance policy effectiveness and acceptance after COVID-19-related restrictions. This is especially important as tailored interventions with public engagement have changed since COVID-19. New engagement strategies working with community leaders, groups, and stakeholders, healthcare groups, and residents may be necessary to overcome challenges related to mistrust, confusion, and limitations for new programs in many communities [25].

The results of the present study should be interpreted with two primary limitations. First, the current study includes a convenience sample of DCHA properties and participants and may not generalize to all DCHA properties or to other public housing authorities in the US. A second limitation is that descriptive results identified demographic differences (age, sex, and education) across the time points. These characteristics have mixed results to support a smoke-free housing rule in prior studies [3,11,17], and current regression models account for these differences with no significant findings. Additional study may be necessary to investigate demographic differences in support for the mandatory rule.

## 5. Conclusions

Despite these limitations, this study provides important longer-term information about support following the implementation of the federally mandated smoke-free housing rule. Results showed an increase in support among smokers following implementation typical of other smoke-free rules, including rules affecting housing, but a slight drop during COVID-19 restrictions. Interventions to continue to increase support, both community-wide and among smokers, may be useful. This may include increasing outreach and cessation support to help smokers comply with these rules.

## Figures and Tables

**Table 1:** Sample characteristics and smoke-free rule support across data collection period (*n* = 529).

Characteristic	Overall % (*n*)	Pre-Rule (T1)	Implementation (T2)	COVID-19 (T3)	Chi-Squared *p*-Value
			
Total	529	12.3% (65)	69.8% (369)	18.0% (95)	
**Age**					<0.01
18–64	66.5% (352)	83.1% (54)	62.9% (232)	69.5% (66)	
65 and older	33.5% (177)	16.9% (11)	37.1% (137)	30.5% (29)	
**Sex**					<0.01
Female	61.6% (326)	76.9% (50)	56.1% (207)	72.6% (69)	
Male	38.4% (203)	23.1% (15)	43.9% (162)	27.4% (26)	
**Highest Education**					<0.01
Less than high school	43.5% (230)	46.2% (30)	46.6% (172)	29.5% (28)	
High school diploma or more	56.5% (299)	53.9% (35)	53.4% (197)	70.5% (67)	
**Supports the Smoke-Free Rule**					0.01
Yes	37.4% (198)	23.1% (15)	41.2% (152)	32.6% (31)	
No	62.6% (331)	76.9% (50)	58.8% (217)	67.4% (64)	
**Tobacco User**					0.11
Yes	50.5% (267)	58.5% (38)	51.2% (189)	41.1% (40)	
No	49.5% (262)	41.5% (27)	48.8% (180)	57.9% (55)	
**Supports the Smoke-Free Rule**					
Tobacco User		5.3% (2)	30.7% (58)	22.5% (9)	<0.01
Non-Tobacco User		48.2% (13)	52.2% (94)	40.0% (22)	0.28

**Table 2:** The association between resident characteristics and support for the smoke-free rule, unstratified and stratified models (*n* = 529).

	Unstratified Model	Stratified Models	
		Tobacco Users	Tobacco Non-Users
	aOR (95% CI)	aOR (95% CI)	aOR (95% CI)
Total	529	267	262
**Time Cluster**			
Pre-rule (T1)	0.47 (0.25, 0.90)	0.14 (0.03, 0.59)	0.94 (0.41, 2.14)
Implementation (T2)	ref	ref	ref
COVID-19 (T3)	0.65 (0.39, 1.07)	0.69 (0.30, 1.56)	0.64 (0.34, 1.19)
**Sex (male)**	1.30 (0.88, 1.92)	1.57 (0.88, 2.80)	1.11 (0.65, 1.89)
**Age**	1.10 (0.74, 1.62)	0.85 (0.45, 1.62)	1.32 (0.79, 2.21)
**Education**	0.98 (0.65, 1.41)	0.94 (0.53, 1.67)	1.02 (0.62, 1.69)
**Tobacco Use Status**	0.34 (0.23, 0.50)		

## Data Availability

The data used to support the findings of this study are available from the corresponding author upon request.
